# Supplementary material for: Inspiring Anti-Tick Vaccine Research, Development and Deployment in Tropical Africa for the Control of Cattle Ticks: Review and Insights
Source: Vaccines (Basel). 2022 Dec 31;11(1):99. doi: 10.3390/vaccines11010099 (PMC9866923; doi:10.3390/vaccines11010099)
Supplement: Supplementary file 1 [file vaccines-11-00099-s001.zip › vaccines-2085243-supplementary.pdf]

Table S1. Selected anti-tick antigens (ATAs) and how they score against some of the proposed characteristics of an ideal ATA.

Antigen (Ag)	Exposed or concealed antigen	Host antibody sufficient access target protein	Antibody-antigen reaction affects tick biology	Conserved epitopes hence protection against multiple species	Ag-coding gene expressed in all tick instars	Ag-coding gene expressed in various tissues	Ag-coding gene expressed in different physiologic states	Ab-Ag reaction affects various Physiologic functions	Protection against pathogen transmission	Efficacy (%) and Tick species challenged	References
Bm86/Bm95	Concealed	✓	✓		✓		✓	✓		45 – 100% Feeding and reproduction	[24, 25, 42, 51, 53]
Subolesin (SUB)	Concealed	✓	✓		✓	✓	✓	✓	✓	1-80% Feeding and reproduction	[26, 33, 45, 148]
<u>Tick feeding</u>											
p29	Exposed	✓	✓		✓						
HL34	Exposed	✓	✓		✓	✓	✓			40-55% Mortality	
RIM36	Exposed	✓	✓		✓		✓	✓			[40, 64 – 68]
64TRPs	Both	✓	✓	✓	✓	✓	✓	✓		47- 62% Mortality	

<u>Haemoglobinolytic enzymes</u><br>Aspartic and cysteine peptidases (cathepsins D, L, B & C types), Asparaginyl endopeptidases, Monopeptidases	Concealed	✓	✓		✓			✓			[9, 85, 93 – 97]
<u>Haeme & Iron transport and storage</u><br>HeLp	Concealed	✓	✓	✓	✓			✓			
Ferritins 1	Concealed		✓	✓	✓			✓			[24, 103, 104, 106, 108, 109, 110]
Ferritins 2	Concealed	✓	✓	✓	✓		✓	✓		50-72% efficacy	
<u>Metalloproteases</u><br>Metis 1 and 2<br>HLMP1	Exposed	✓	✓		✓	✓		✓	✓	14-60% Engorgement, oviposition, fertility & mortality	[23, 26, 63, 127 – 134]
<u>Protease inhibitors</u><br><u>Serpins;</u> HL S2, RA S 1-4	Both	✓	✓		✓	✓		✓		28-43%	

<u>Cystatins:</u>											
Bmcystatin, Hlcyst-I	Both	✓	✓		✓			✓		Engorgement & mortality 40%	[60, 71 -84, 86]
Sialostatin L(SL), L2 (SL2), Hlcyst-2 and -3, HLSC-I, om-cystatin I, 2	Exposed	✓	✓		✓			✓	✓	Attachment & feeding	
<u>Detoxification</u>											
Glutathione S-transferases (GST)	Concealed	✓	✓	✓	✓	✓	✓	✓		50-57% Efficacy	[55, 111, 113, 115, 116]
<u>Osmoregulation</u>											
Aquaporins: RmAQP1, 2&3	Concealed	✓	✓	✓	✓		✓			68-75% Efficacy	[88 – 92]
<u>Reproduction:</u>											
Boophilus yolk pro-cathepsin (BYC)	Concealed	✓	✓	✓	✓	✓		✓		25-36% Tick number, weight& fertility	[44, 117 – 125]
Tick haeme-binding aspartic peptidase (*THAP)*	Concealed	✓	✓	✓	✓			✓			
Vitellin degrading cysteine endo-peptidase (VTDCE)	Concealed	✓	✓	✓	✓			✓		21% Egg weight, No. engorged	
<u>Mating</u>											
Engorgement factor (voraxin) e.g., rAhEF	Concealed	✓	✓					✓	✓	72% Tick weight	[135, 136]

References

1. de la Fuente, J., & Contreras, M. (2015). Tick vaccines: Current status and future directions. Expert Review of Vaccines, 14(10), 1367–1376. https://doi.org/10.1586/14760584.2015.1076339

2. Jaime Betancur Hurtado, O., & Giraldo-Ríos, C. (2019). Economic and Health Impact of the Ticks in Production Animals. Ticks and Tick-Borne Pathogens. https://doi.org/10.5772/intechopen.81167

3. Githaka, N. W., Kanduma, E. G., Wieland, B., Darghouth, M. A., & Bishop, R. P. (2022). Acaricide resistance in livestock ticks infesting cattle in Africa: Current status and potential mitigation strategies. Current Research in Parasitology & Vector-Borne Diseases, 2(February), 100090. https://doi.org/10.1016/j.crpvbd.2022.100090

4. Kerario, I. I., Simuunza, M., Laisser, E. L. K., & Chenyambuga, S. (2018). Exploring knowledge and management practices on ticks and tick-borne diseases among agro-pastoral communities in Southern Highlands, Tanzania. Veterinary World, 11(1), 48–57. https://doi.org/10.14202/vetworld.2018.48-57

5. Jongejan, F., & Uilenberg, G. (2004). The global importance of ticks. In Parasitology (Vol. 129, Issue SUPPL.). https://doi.org/10.1017/S0031182004005967

6. de la Fuente, José. (2012). Vaccines for vector control: exciting possibilities for the future. Veterinary Journal, 194(2), 139–140. https://doi.org/10.1016/j.tvjl.2012.07.029

7. Kasaija, P. D., Estrada-Peña, A., Contreras, M., Kirunda, H., & de la Fuente, J. (2021). Cattle ticks and tick-borne diseases: a review of Uganda's situation. Ticks and Tick-Borne Diseases, 12(5). https://doi.org/10.1016/j.ttbdis.2021.101756

8. Young, A. S., Groocock, C. M., & Kariuki, D. P. (1988). No Title. Parasitology, 96(02), 403. https://doi.org/10.1017/s0031182000058388

9. Parizi, L. F., Pohl, P. C., Masuda, A., & Junior, I. D. S. V. (2009). New approaches toward anti-Rhipicephalus (Boophilus) microplus tick vaccine. Revista Brasileira de Parasitologia Veterinária, 18(01), 1–7. https://doi.org/10.4322/rbpv.01801001

10. Ghosh, S., Azhahianambi, P., & Yadav, M. P. (2007). Upcoming and future strategies of tick control: A review. Journal of Vector Borne Diseases, 44(2), 79–89.

11. Klafke, G. M., Sabatini, G. A., de Albuquerque, T. A., Martins, J. R., Kemp, D. H., Miller, R. J., & Schumaker, T. T. S. (2006). Larval immersion tests with ivermectin in populations of the cattle tick Rhipicephalus (Boophilus) microplus (Acari: Ixodidae) from State of Sao Paulo, Brazil. Veterinary Parasitology, 142(3–4), 386–390. https://doi.org/10.1016/j.vetpar.2006.07.001

12. de la Fuente, J., Rodríguez, M., Montero, C., Redondo, M., García-García, J. C., Méndez, L., Serrano, E., Valdés, M., Enríquez, A., Canales, M., Ramos, E., Boué, O., Machado, H., & Lleonart, R. (1999). Vaccination against ticks (Boophilus spp.): The experience with the Bm86-based vaccine Gavac((TM)). Genetic Analysis - Biomolecular Engineering, 15(3–5), 143–148. https://doi.org/10.1016/S1050-3862(99)00018-2

13. Willadsen, Peter, Smith, D., Cobon, G., & McKenna, R. V. (1996). Comparative vaccination of cattle against *Boophilus microplus* with recombinant antigen Bm86 alone or in combination with recombinant Bm91. Parasite Immunology, 18(5), 241–246. https://doi.org/10.1046/j.1365-3024.1996.d01-90.x

14. Gasparin, G., Miyata, M., Coutinho, L. L., Martinez, M. L., Teodoro, R. L., Furlong, J., Machado, M. A., Silva, M. V. G. B., Sonstegard, T. S., & Regitano, L. C. A. (2007). Mapping of quantitative trait loci controlling tick

[Riphicephalus (Boophilus) microplus] resistance on bovine chromosomes 5, 7 and 14. Animal Genetics, 38(5), 453–459. https://doi.org/10.1111/j.1365-2052.2007.01634.x

15. Mapholi, N. O., Marufu, M. C., Maiwashe, A., Banga, C. B., Muchenje, V., MacNeil, M. D., Chimonyo, M., & Dzama, K. (2014). Towards a genomics approach to tick (Acari: Ixodidae) control in cattle: A review. Ticks and Tick-Borne Diseases, 5(5), 475–483. https://doi.org/10.1016/j.ttbdis.2014.04.006

16. Byaruhanga, C., Collins, N. E., Knobel, D., Kabasa, W., & Oosthuizen, M. C. (2016). Veterinary Parasitology: Regional Studies and Reports Endemic status of tick-borne infections and tick species diversity among transhumant zebu cattle in Karamoja Region, Uganda: Support for control approaches. VPRSR, 1–10. https://doi.org/10.1016/j.vprsr.2015.11.001

17. Vudriko, P., Okwee-Acai, J., Tayebwa, D. S., Byaruhanga, J., Kakooza, S., Wampande, E., Omara, R., Muhindo, J. B., Tweyongyere, R., Owiny, D. O., Hatta, T., Tsuji, N., Umemiya-Shirafuji, R., Xuan, X., Kanameda, M., Fujisaki, K., & Suzuki, H. (2016). Emergence of multi-acaricide resistant Rhipicephalus ticks and its implication on chemical tick control in Uganda. Parasites and Vectors, 9(1). https://doi.org/10.1186/s13071-015-1278-3

18. Chenyambuga, S. W., Waiswa, C., Saimo, M., Ngumi, P., & Gwakisa, P. S. (2010). Knowledge and perceptions of traditional livestock keepers on tick-borne diseases and sero-prevalence of Theileria parva around Lake Victoria Basin. Livestock Research for Rural Development, 22(7).

19. Mugisha, A., McLeod, A., Percy, R., & Kyewalabye, E. (2008). Socio-economic factors influencing control of vector-borne diseases in the pastoralist system of south western Uganda. Tropical Animal Health and Production, 40(4), 287–297. https://doi.org/10.1007/s11250-007-9093-2

20. Tayebwa, D. S., Vudriko, P., Tuvshintulga, B., Guswanto, A., Nugraha, A. B., Gantuya, S., Batiha, G. E. S., Musinguzi, S. P., Komugisha, M., Bbira, J. S., Okwee-Acai, J., Tweyongyere, R., Wampande, E. M., Byaruhanga, J., Adjou Moumouni, P. F., Sivakumar, T., Yokoyama, N., & Igarashi, I. (2018). Molecular epidemiology of Babesia species, Theileria parva, and Anaplasma marginale infecting cattle and the tick control malpractices in Central and Eastern Uganda. Ticks and Tick-Borne Diseases, 9(6), 1475–1483. https://doi.org/10.1016/j.ttbdis.2018.06.012

21. Vudriko, P., Okwee-Acai, J., Byaruhanga, J., Tayebwa, D. S., Okech, S. G., Tweyongyere, R., Wampande, E. M., Okurut, A. R. A., Mugabi, K., Muhindo, J. B., Nakavuma, J. L., Umemiya-Shirafuji, R., Xuan, X., & Suzuki, H. (2018). Chemical tick control practices in southwestern and northwestern Uganda. Ticks and Tick-Borne Diseases, 9(4), 945–955. https://doi.org/10.1016/j.ttbdis.2018.03.009

22. Abbas, R. Z., Zaman, M. A., Colwell, D. D., Gilleard, J., & Iqbal, Z. (2014). Acaricide resistance in cattle ticks and approaches to its management: The state of play. Veterinary Parasitology, 203(1–2), 6–20. https://doi.org/10.1016/j.vetpar.2014.03.006

23. Ali, A., Fernando Parizi, L., Garcia Guizzo, M., Tirloni, L., Seixas, A., Silva Vaz, I. da, & Termignoni, C. (2015). Immunoprotective potential of a Rhipicephalus (Boophilus) microplus metalloprotease. Veterinary Parasitology, 207(1–2), 107–114. https://doi.org/10.1016/j.vetpar.2014.11.007

24. Hajdusek, O., Almazán, C., Loosova, G., Villar, M., Canales, M., Grubhoffer, L., Kopacek, P., & de la Fuente, J. (2010a). Characterization of ferritin 2 for the control of tick infestations. Vaccine, 28(17), 2993–2998. https://doi.org/10.1016/j.vaccine.2010.02.008

25. de la Fuente, José, Kocan, K. M., Almazán, C., & Blouin, E. F. (2007b). RNA interference for the study and genetic manipulation of ticks. Trends in Parasitology, 23(9), 427–433. https://doi.org/10.1016/j.pt.2007.07.002

26. Merino, O., Almazán, C., Canales, M., Villar, M., Moreno-Cid, J. A., Galindo, R. C., & De la Fuente, J. (2011). Targeting the tick protective antigen subolesin reduces vector infestations and pathogen infection by Anaplasma marginale and Babesia bigemina. Vaccine, 29(47), 8575–8579. https://doi.org/10.1016/j.vaccine.2011.09.023

27. de la Fuente, José, Almazán, C., Canales, M., Pérez de la Lastra, J. M., Kocan, K. M., & Willadsen, P. (2007a). A ten-year review of commercial vaccine performance for control of tick infestations on cattle. Animal Health Research Reviews / Conference of Research Workers in Animal Diseases, 8(1), 23–28. https://doi.org/10.1017/S1466252307001193

28. Willadsen, Peter. (2008). Antigen cocktails: valid hypothesis or unsubstantiated hope? Trends in Parasitology, 24(4), 164–167. https://doi.org/10.1016/j.pt.2008.01.005

29. de la Fuente, José, & Estrada-Peña, A. (2019a). Why new vaccines for the control of ectoparasite vectors have not been registered and commercialized? In Vaccines (Vol. 7, Issue 3, pp. 11–15). https://doi.org/10.3390/vaccines7030075

30. Parizi, Luís F., Githaka, N. W., Logullo, C., Konnai, S., Masuda, A., Ohashi, K., & da Silva Vaz, I. (2012a). The quest for a universal vaccine against ticks: Cross-immunity insights. Veterinary Journal, 194(2), 158–165. https://doi.org/10.1016/j.tvjl.2012.05.023

31. Cunha, R. C., Perez de Leon, A. A., Leite, F. P. L., Pinto, L. da S., Dos Santos Junior, A. G., & Andreotti, R. (2012). Bovine immunoprotection against Rhipicephalus (Boophilus) microplus with recombinant Bm86-Campo Grande antigen. Revista Brasileira de Parasitologia Veterinaria, 21(3), 254–262. https://doi.org/10.1590/s1984-29612012000300014

32. Odongo, D., Kamau, L., Skilton, R., Mwaura, S., Nitsch, C., Musoke, A., Taracha, E., Daubenberger, C., & Bishop, R. (2007). Vaccination of cattle with TickGARD induces cross-reactive antibodies binding to conserved linear peptides of Bm86 homologues in Boophilus decoloratus. Vaccine, 25(7), 1287–1296. https://doi.org/10.1016/j.vaccine.2006.09.085

33. Moreno-cid, J. (2013). Control of multiple arthropod vector infestations with subolesin akirin vaccines. August. https://doi.org/10.1016/j.vaccine.2012.12.073

34. Almazán, C., Moreno-Cantú, O., Moreno-Cid, J. A., Galindo, R. C., Canales, M., Villar, M., & De la Fuente, J. (2012). Control of tick infestations in cattle vaccinated with bacterial membranes containing surface-exposed tick protective antigens. Vaccine, 30(2), 265–272. https://doi.org/10.1016/j.vaccine.2011.10.102

35. Torina, A., Moreno-Cid, J. A., Blanda, V., Fernández De Mera, I. G., De La Lastra, J. M. P., Scimeca, S., Blanda, M., Scariano, M. E., Briganò, S., Disclafani, R., Piazza, A., Vicente, J., Gortázar, C., Caracappa, S., Lelli, R. C., & De La Fuente, J. (2014). Control of tick infestations and pathogen prevalence in cattle and sheep farms vaccinated with the recombinant Subolesin-Major Surface Protein 1a chimeric antigen. Parasites and Vectors, 7(1), 1–15. https://doi.org/10.1186/1756-3305-7-10

36. Merino, O., Alberdi, P., Pérez De La Lastra, J. M., & de la Fuente, J. (2013). Tick vaccines and the control of tick-borne pathogens. Frontiers in Cellular and Infection Microbiology, 4(JUL), 1–10. https://doi.org/10.3389/fcimb.2013.00030

37. García-García, J. C., Montero, C., Redondo, M., Vargas, M., Canales, M., Boue, O., Rodríguez, M., Joglar, M., MacHado, H., González, I. L., Valdés, M., Méndez, L., & De La Fuente, J. (2000). Control of ticks resistant to immunization with Bm86 in cattle vaccinated with the recombinant antigen Bm95 isolated from the cattle tick, *Boophilus microplus*. Vaccine, 18(21), 2275–2287. https://doi.org/10.1016/S0264-410X(99)00548-4

38. Allen, J. R., & Humphreys, S. J. (1979). Immunization of Guinea pigs and cattle against ticks. In Nature (Vol. 280, pp. 491–493).

39. Roberts, J. A. (1968). Resistance of Cattle to the Tick *Boophilus microplus* (Canestrini). I. Development of Ticks on Bos taurus. The Journal of Parasitology, 54(4), 663. https://doi.org/10.2307/3277015

40. Bishop, R., Lambson, B., Wells, C., Pandit, P., Osaso, J., Nkonge, C., Morzaria, S., Musoke, A., & Nene, V. (2002). A cement protein of the tick *Rhipicephalus appendiculatus*, located in the secretory e cell granules of the type III salivary gland acini, induces strong antibody responses in cattle. International Journal for Parasitology, 32(7), 833–842. https://doi.org/10.1016/S0020-7519(02)00027-9

41. Masood Akhtar, Faqir Muhammad, Laeeq Akbar Lodhi, I. H. and M. I. A. (2010). Pakistan Veterinary Journal. Animals, 8318(2), 85–92. https://doi.org/10.1097/QCO.0b013e3283638104

42. de la Fuente, J., Rodríguez, M., Redondo, M., Montero, C., García-García, J. C., Méndez, L., Serrano, E., Valdés, M., Enriquez, A., Canales, M., Ramos, E., Boué, O., Machado, H., Lleonart, R., De Armas, C. A., Rey, S., Rodríguez, J. L., Artiles, M., & García, L. (1998). Field studies and cost-effectiveness analysis of vaccination with Gavac(TM) against the cattle tick *Boophilus microplus*. Vaccine, 16(4), 366–373. https://doi.org/10.1016/S0264-410X(97)00208-9

43. Canales, M., Enríquez, A., Ramos, E., Cabrera, D., Dandie, H., Soto, A., Falcón, V., Rodríguez, M., & De la Fuente, J. (1997). Large-scale production in Pichia pastoris of the recombinant vaccine Gavac(TM) against cattle tick. Vaccine, 15(4), 414–422. https://doi.org/10.1016/S0264-410X(96)00192-2

44. Brake, D. K., & Pérez De León, A. A. (2012). Immunoregulation of bovine macrophages by factors in the salivary glands of Rhipicephalus microplus. Parasites and Vectors, 5(1), 1–8. https://doi.org/10.1186/1756-3305-5-38

45. de la Fuente, Jose, Contreras, M., Kasaija, P. D., Gortazar, C., Ruiz-Fons, J. F., Mateo, R., & Kabi, F. (2019b). Towards a Multidisciplinary Approach to Improve Cattle Health and Production in Uganda. In Vaccines (Vol. 7, Issue 4). https://doi.org/10.3390/vaccines7040165

46. Elvin, C. M., & Kemp, D. H. (1994). Generic approaches to obtaining efficacious antigens from vector arthropods. International Journal for Parasitology, 24(1), 67–79. https://doi.org/10.1016/0020-7519(94)90060-4

47. Trimnell, A. R., Kazimírová, M., Klempa, B., Havlíková, S., Roller, L., Koc, J., & Nuttall, P. A. (2009). Functional role of 64P , the candidate transmission-blocking vaccine antigen from the tick , *Rhipicephalus appendiculatus* q. 39, 1485–1494. https://doi.org/10.1016/j.ijpara.2009.05.005

48. Willadsen, P, Riding, G. A., McKenna, R. V, Kemp, D. H., Tellam, R. L., Nielsen, J. N., Lahnstein, J., Cobon, G. S., & Gough, J. M. (1989). Immunologic control of a parasitic arthropod. Identification of a protective antigen from *Boophilus microplus*. Journal of Immunology (Baltimore, Md. : 1950), 143(4), 1346–1351. http://www.ncbi.nlm.nih.gov/pubmed/2745982

49. Willadsen, P., McKenna, R. V., & Riding, G. A. (1988). Isolation from the cattle tick, *Boophilus microplus*, of antigenic material capable of eliciting a protective immunological response in the bovine host. International Journal for Parasitology, 18(2), 183–189. https://doi.org/10.1016/0020-7519(88)90059-8

50. Valle, M. R., Mèndez, L., Valdez, M., Redondo, M., Espinosa, C. M., Vargas, M., Cruz, R. L., Barrios, H. P., Seoane, G., Ramirez, E. S., Boue, O., Vigil, J. L., Machado, H., Nordelo, C. B., & Piñeiro, M. J. (2004). Integrated control of *Boophilus microplus* ticks in Cuba based on vaccination with the anti-tick vaccine GavacTM. Experimental and Applied Acarology, 34(3–4), 375–382. https://doi.org/10.1007/s10493-004-1389-6

51. de la Fuente, José, & Kocan, K. M. (2003). Advances in the identification and characterization of protective antigens for recombinant vaccines against tick infestations. Expert Review of Vaccines, 2(4), 583–593. https://doi.org/10.1586/14760584.2.4.583

52. de Vos, S., Zeinstra, L., Taoufik, O., Willadsen, P., & Jongejan, F. (2001). Evidence for the utility of the Bm86 antigen from *Boophilus microplus* in vaccination against other tick species. Experimental & Applied Acarology, 25(3), 245–261. https://doi.org/10.1023/a:1010609007009

53. Canales, M., Almazán, C., Naranjo, V., Jongejan, F., & de la Fuente, J. (2009). Vaccination with recombinant *Boophilus annulatus* Bm86 ortholog protein, Ba86, protects cattle against B. annulatus and B. microplus infestations. BMC Biotechnology, 9, 1–8. https://doi.org/10.1186/1472-6750-9-29

54. de la Fuente, J., Kopáček, P., Lew-Tabor, A., & Maritz-Olivier, C. (2016). Strategies for new and improved vaccines against ticks and tick-borne diseases. Parasite Immunology, 38(12), 754–769. https://doi.org/10.1111/pim.12339

55. Anderson, J. F., & Magnarelli, L. A. (2008). Biology of Ticks. 22, 195–215. https://doi.org/10.1016/j.idc.2007.12.006

56. Kotál, J., Langhansová, H., Lieskovská, J., Andersen, J. F., Francischetti, I. M. B., Chavakis, T., Kopecký, J., Pedra, J. H. F., Kotsyfakis, M., & Chmelař, J. (2015). Modulation of host immunity by tick saliva. Journal of Proteomics, 128, 58–68. https://doi.org/10.1016/j.jprot.2015.07.005

57. Valenzuela, J. G., Belkaid, Y., Garfield, M. K., Mendez, S., Kamhawi, S., Rowton, E. D., Sacks, D. L., & Ribeiro, J. M. C. (2001). Toward a defined anti-Leishmania vaccine targeting vector antigens: Characterization of a protective salivary protein. *Journal of Experimental Medicine*, *194*(3), 331–342. https://doi.org/10.1084/jem.194.3.331

58. Maharana, B. R., Baithalu, R. K., Allaie, I. M., Mishra, C., & Samal, L. (2011). Mechanism of immunity to tick infestation in livestock. Veterinary World, 4(3), 131–135. https://doi.org/10.5455/vetworld.2011.131-135

59. Wikel, S. K. (2018). Tick-host-pathogen systems immunobiology: an interactive Trio 2. Introduction to Ticks, Host Interactions and Tick-Borne Diseases. 265–283.

60. Kazimírová, M., & Štibrániová, I. (2013). Tick salivary compounds: Their role in modulation of host defences and pathogen transmission. Frontiers in Cellular and Infection Microbiology, 4(AUG), 1–19. https://doi.org/10.3389/fcimb.2013.00043

61. Hermance, M. E., & Thangamani, S. (2014). Proinflammatory cytokines and chemokines at the skin interface during powassan virus transmission. Journal of Investigative Dermatology, 134(8), 2280–2283. https://doi.org/10.1038/jid.2014.150

62. Hermance, M. E., & Thangamani, S. (2015). Tick Saliva Enhances Powassan Virus Transmission to the Host, Influencing Its Dissemination and the Course of Disease. Journal of Virology, 89(15), 7852–7860. https://doi.org/10.1128/jvi.01056-15

63. Mulenga, Albert, Sugimoto, C., Sako, Y., Ohashi, K., Musoke, A., Shubash, M., & Onuma, M. (1999). Molecular characterization of a *Haemaphysalis longicornis* tick salivary gland-associated 29-kilodalton protein and its effect as a vaccine against tick infestation in rabbits. Infection and Immunity, 67(4), 1652–1658. https://doi.org/10.1128/iai.67.4.1652-1658.1999

64. Tsuda, A., Mulenga, A., Sugimoto, C., Nakajima, M., Ohashi, K., & Onuma, M. (2001). cDNA cloning, characterization and vaccine effect analysis of *Haemaphysalis longicornis* tick saliva proteins. Vaccine, 19(30), 4287–4296. https://doi.org/10.1016/S0264-410X(01)00148-7

65. Shapiro, S. Z., Voigt, W. P., & Fujisaki, K. (1986). Tick antigens recognized by serum from a guinea pig resistant to infestation with the tick *Rhipicephalus appendiculatus*. Journal of Parasitology, 72(3), 454–463. https://doi.org/10.2307/3281686

66. Trimnell, A. R., Davies, G. M., Lissina, O., Hails, R. S., & Nuttall, P. A. (2005). A cross-reactive tick cement antigen is a candidate broad-spectrum tick vaccine. Vaccine, 23(34), 4329–4341. https://doi.org/10.1016/j.vaccine.2005.03.041

67. Trimnell, A. R., Hails, R. S., & Nuttall, P. A. (2002). Dual action ectoparasite vaccine targeting "exposed" and "concealed" antigens. Vaccine, 20(29–30), 3560–3568. https://doi.org/10.1016/S0264-410X(02)00334-1

68. Barriga, O. O. (1999). Evidence and mechanisms of immunosuppression in tick infestations. Genetic Analysis - Biomolecular Engineering, 15(3–5), 139–142. https://doi.org/10.1016/S1050-3862(99)00017-0

69. Wikel, S. K. (1996). Host immunity to ticks. Annual Review of Entomology, 41(1), 1–22. https://doi.org/10.1146/annurev.en.41.010196.000245

70. Gettins PGW. (1996). Serpins: structure, function and biology. In Springer. Chapman and Hall.

71. Rubin H. (1996). Serine protease inhibitors (SERPINS): where mechanism meets medicine. 2(6), 632–633.

72. Imamura, S., da Silva Vaz Junior, I., Sugino, M., Ohashi, K., & Onuma, M. (2005). A serine protease inhibitor (serpin) from *Haemaphysalis longicornis* as an anti-tick vaccine. Vaccine, 23(10), 1301–1311. https://doi.org/10.1016/j.vaccine.2004.08.041

73. Andreotti, R., Gomes, A., Malavazi-piza, K. C., Sasaki, S. D., Sampaio, C. A. M., & Tanaka, A. S. (2002). BmTI antigens induce a bovine protective immune response against *Boophilus microplus* tick. 2, 557–563.

74. Sugino, M., Imamura, S., Mulenga, A., Nakajima, M., Tsuda, A., Ohashi, K., & Onuma, M. (2003). A serine proteinase inhibitor (serpin) from ixodid tick Haemaphysalis longicornis; cloning and preliminary assessment of its suitability as a candidate for a tick vaccine. Vaccine, 21(21–22), 2844–2851. https://doi.org/10.1016/s0264-410x(03)00167-1

75. Mulenga, A., Tsuda, A., Onuma, M., & Sugimoto, C. (2003). Four serine proteinase inhibitors (serpin) from the brown ear tick, *Rhiphicephalus appendiculatus*; cDNA cloning and preliminary characterization. Insect Biochemistry and Molecular Biology, 33(2), 267–276. https://doi.org/10.1016/S0965-1748(02)00240-0

76. Imamura, S., Namangala, B., Tajima, T., Tembo, M. E., Yasuda, J., Ohashi, K., & Onuma, M. (2006). Two serine protease inhibitors (serpins) that induce a bovine protective immune response against *Rhipicephalus appendiculatus* ticks. Vaccine, 24(13), 2230–2237. https://doi.org/10.1016/j.vaccine.2005.10.055

77. Lima, C. A., Sasaki, S. D., & Tanaka, A. S. (2006). Bmcystatin, a cysteine proteinase inhibitor characterized from the tick *Boophilus microplus*. Biochemical and Biophysical Research Communications, 347(1), 44–50. https://doi.org/10.1016/j.bbrc.2006.06.018

78. Zavašnik-Bergant, T., & Turk, B. (2006). Cysteine cathepsins in the immune response. Tissue Antigens, 67(5), 349–355. https://doi.org/10.1111/j.1399-0039.2006.00585.x

79. Kotsyfakis, M., Karim, S., Andersen, J. F., Mather, T. N., & Ribeiro, J. M. C. (2007). Selective cysteine protease inhibition contributes to blood-feeding success of the tick Ixodes scapularis. The Journal of Biological Chemistry, 282(40), 29256–29263. https://doi.org/10.1074/jbc.M703143200

80. Kotsyfakis, M., Anderson, J. M., Andersen, J. F., Calvo, E., Francischetti, I. M. B., Mather, T. N., Valenzuela, J. G., & Ribeiro, J. M. C. (2008). Cutting Edge: Immunity against a "Silent" Salivary Antigen of the Lyme Vector Ixodes scapularis Impairs Its Ability to Feed. The Journal of Immunology, 181(8), 5209–5212. https://doi.org/10.4049/jimmunol.181.8.5209

81. Yamaji, K., Tsuji, N., Miyoshi, T., Islam, M. K., Hatta, T., Alim, M. A., Anisuzzaman, M., Kushibiki, S., & Fujisaki, K. (2009b). A salivary cystatin, HlSC-1, from the ixodid tick *Haemaphysalis longicornis* play roles in the blood-feeding processes. Parasitology Research, 106(1), 61–68. https://doi.org/10.1007/s00436-009-1626-3

82. Zhou, J., Ueda, M., Umemiya, R., Battsetseg, B., Boldbaatar, D., Xuan, X., & Fujisaki, K. (2006). A secreted cystatin from the tick *Haemaphysalis longicornis* and its distinct expression patterns in relation to innate immunity. Insect Biochemistry and Molecular Biology, 36(7), 527–535. https://doi.org/10.1016/j.ibmb.2006.03.003

83. Schwarz, A., Valdés, J. J., & Kotsyfakis, M. (2012). The role of cystatins in tick physiology and blood feeding. Ticks and Tick-Borne Diseases, 3(3), 117–127. https://doi.org/10.1016/j.ttbdis.2012.03.004

84. Yamaji, K., Tsuji, N., Miyoshi, T., Islam, M. K., Hatta, T., Alim, M. A., Anisuzzaman, Takenaka, A., & Fujisaki, K. (2009a). Hemoglobinase activity of a cysteine protease from the ixodid tick Haemaphysalis longicornis. Parasitology International, 58(3), 232–237. https://doi.org/10.1016/j.parint.2009.05.003

85. Grunclová, L., Horn, M., Vancová, M., Sojka, D., Franta, Z., Mareš, M., & Kopáček, P. (2006). Two secreted cystatins of the soft tick Ornithodoros moubata: Differential expression pattern and inhibitory specificity. Biological Chemistry, 387(12), 1635–1644. https://doi.org/10.1515/BC.2006.204

86. Salát, J., Paesen, G. C., Řezáčová, P., Kotsyfakis, M., Kovářová, Z., Šanda, M., Majtán, J., Grunclová, L., Horká, H., Andersen, J. F., Brynda, J., Horn, M., Nunn, M. A., Kopáček, P., Kopecký, J., & Mareš, M. (2010). Crystal structure and functional characterization of an immunomodulatory salivary cystatin from the soft tick Ornithodoros moubata. Biochemical Journal, 429(1), 103–112. https://doi.org/10.1042/BJ20100280

87. Bowman, A. S., & Sauer, J. R. (2004). Tick salivary glands: Function, physiology and future. Parasitology, 129(SUPPL.). https://doi.org/10.1017/S0031182004006468

88. Megaw, M. W. J. (1974). Studies on the water balance mechanism of the tick, *Boophilus microplus* canestrini. Comparative Biochemistry and Physiology -- Part A: Physiology, 48(1), 115–125. https://doi.org/10.1016/0300-9629(74)90859-7

89. Valenzuela, J. G. (2004). Exploring tick saliva: From biochemistry to "sialomes" and functional genomics. Parasitology, 129(SUPPL.). https://doi.org/10.1017/S0031182004005189

90. Campbell, E. M., Ball, A., Hoppler, S., & Bowman, A. S. (2008). Invertebrate aquaporins: A review. Journal of Comparative Physiology B: Biochemical, Systemic, and Environmental Physiology, 178(8), 935–955. https://doi.org/10.1007/s00360-008-0288-2

91. Guerrero, F. D., Andreotti, R., Bendele, K. G., Cunha, R. C., Miller, R. J., Yeater, K., & De León, A. A. P. (2014). Rhipicephalus (Boophilus) microplus aquaporin as an effective vaccine antigen to protect against cattle tick infestations. Parasites and Vectors, 7(1), 1–12. https://doi.org/10.1186/s13071-014-0475-9

92. Sojka, D., Franta, Z., Horn, M., Caffrey, C. R., Mareš, M., & Kopáček, P. (2013). New insights into the machinery of blood digestion by ticks. Trends in Parasitology, 29(6), 276–285. https://doi.org/10.1016/j.pt.2013.04.002

93. Franta, Z., Sojka, D., Frantova, H., Dvorak, J., Horn, M., Srba, J., Talacko, P., Mares, M., Schneider, E., Craik, C. S., McKerrow, J. H., Caffrey, C. R., & Kopacek, P. (2011). IrCL1 - The haemoglobinolytic cathepsin L of the hard tick, Ixodes ricinus. International Journal for Parasitology, 41(12), 1253–1262. https://doi.org/10.1016/j.ijpara.2011.06.006

94. Tsuji, N., Miyoshi, T., Battsetseg, B., Matsuo, T., Xuan, X., & Fujisaki, K. (2008). A cysteine protease is critical for Babesia spp. transmission in Haemaphysalis ticks. PLoS Pathogens, 4(5). https://doi.org/10.1371/journal.ppat.1000062

95. Rudenko, N., Golovchenko, M., Edwards, M. J., & Grubhoffer, L. (2005). Differential expression of Ixodes ricinus tick genes induced by blood feeding or Borrelia burgdorferi infection. Journal of Medical Entomology, 42(1), 36–41. https://doi.org/10.1093/jmedent/42.1.36

96. Horn, M., Nussbaumerová, M., Šanda, M., Kovářová, Z., Srba, J., Franta, Z., Sojka, D., Bogyo, M., Caffrey, C. R., Kopáček, P., & Mareš, M. (2009). Hemoglobin Digestion in Blood-Feeding Ticks: Mapping a Multipeptidase Pathway by Functional Proteomics. Chemistry and Biology, 16(10), 1053–1063. https://doi.org/10.1016/j.chembiol.2009.09.009

97. Lara, F. A., Lins, U., Bechara, G. H., & Oliveira, P. L. (2005). Tracing heme in a living cell: Hemoglobin degradation and heme traffic in digest cells of the cattle tick *Boophilus microplus*. Journal of Experimental Biology, 208(16), 3093–3101. https://doi.org/10.1242/jeb.01749

98. AKOV, S. (1982). Blood Digestion in Ticks. In Physiology of Ticks (Vol. 0). Pergamon Press Ltd. https://doi.org/10.1016/b978-0-08-024937-7.50011-1

99. Renard, G., Lara, F. A., Cardoso, F. C. De, Miguens, F. C., Dansa-petretski, M., Termignoni, C., & Masuda, A. (2002). Expression and immunolocalization of a *Boophilus microplus* cathepsin L-like enzyme. 11(April), 325–328.

100. Lara, Flavio Alves, Lins, U., Paiva-Silva, G., Almeida, I. C., Braga, C. M., Miguens, F. C., Oliveira, P. L., & Dansa-Petretski, M. (2003a). A new intracellular pathway of haem detoxification in the midgut of the cattle tick *Boophilus microplus*: Aggregation inside a specialized organelle, the hemosome. Journal of Experimental Biology, 206(10), 1707–1715. https://doi.org/10.1242/jeb.00334

101. Schmitt, T. H., Frezzatti, W. A., & Schreier, S. (1993). Hemin-Induced Lipid Membrane Disorder and Increased Permeability: A Molecular Model for the Mechanism of Cell Lysis. In Archives of Biochemistry and Biophysics (Vol. 307, Issue 1, pp. 96–103). https://doi.org/10.1006/abbi.1993.1566

102. Braz, G. R. C., Coelho, H. S. L., Masuda, H., & Oliveira, P. L. (1999). A missing metabolic pathway in the cattle tick *Boophilus microplus*. Current Biology, 9(13), 703–706. https://doi.org/10.1016/S0960-9822(99)80312-1

103. Maya-Monteiro, C. M., Daffre, S., Logullo, C., Lara, F. A., Alves, E. W., Capurro, M. L., Zingali, R., Almeida, I. C., & Oliveira, P. L. (2000). HeLp, a heme lipoprotein from the hemolymph of the cattle tick, *Boophilus microplus*. Journal of Biological Chemistry, 275(47), 36584–36589. https://doi.org/10.1074/jbc.M007344200

104. Rosell, R., & Coons, L. B. (1991). Purification and partial characterization of vitellin from the eggs of the hard tick, Dermacentor variabilis. Insect Biochemistry, 21(8), 871–885. https://doi.org/10.1016/0020-1790(91)90094-U

105. Maya-Monteiro, C. M., Alves, L. R., Pinhal, N., Abdalla, D. S. P., & Oliveira, P. L. (2004). HeLp, a heme-transporting lipoprotein with an antioxidant role. Insect Biochemistry and Molecular Biology, 34(1), 81–87. https://doi.org/10.1016/j.ibmb.2003.09.005

106. Rsiþhn, 3hwuu, Burešová, V., & Daffre, S. (2010). Chapter 8 R Tick Innate Immunity. 137–162.

107. Wang, F., Lv, H., Zhao, B., Zhou, L., Wang, S., Luo, J., Liu, J., & Shang, P. (2019). Journal of Experimental and Clinical Cancer Research, 38, 1–17.

108. Galay, R. L., Aung, K. M., Umemiya-Shirafuji, R., Maeda, H., Matsuo, T., Kawaguchi, H., Miyoshi, N., Suzuki, H., Xuan, X., Mochizuki, M., Fujisaki, K., & Tanaka, T. (2013). Multiple ferritins are vital to successful blood feeding and reproduction of the hard tick Haemaphysalis longicornis. Journal of Experimental Biology, 216(10), 1905–1915. https://doi.org/10.1242/jeb.081240

109. Galay, R. L., Umemiya-Shirafuji, R., Bacolod, E. T., Maeda, H., Kusakisako, K., Koyama, J., Tsuji, N., Mochizuki, M., Fujisaki, K., & Tanaka, T. (2014). Two kinds of ferritin protect ixodid ticks from iron overload and consequent oxidative stress. PLoS ONE, 9(3). https://doi.org/10.1371/journal.pone.0090661

110. Parizi, Luís Fernando, Utiumi, K. U., Imamura, S., Onuma, M., Ohashi, K., Masuda, A., & da Silva Vaz, I. (2011). Cross immunity with *Haemaphysalis longicornis* glutathione S-transferase reduces an experimental Rhipicephalus (Boophilus) microplus infestation. Experimental Parasitology, 127(1), 113–118. https://doi.org/10.1016/j.exppara.2010.07.001

111. Sheehan, D. (2001). Structure, function and evolution of glutathione transferases: implications for classification of non-mammalian members of an ancient enzyme superfamily. Biochemical Journal, 360(1), 1–16. https://doi.org/10.1042/0264-6021:3600001

112. Vontas, J. G., Enayati, A. A., Small, G. J., & Hemingway, J. (2000). A simple biochemical assay for glutathione S-transferase activity and its possible field application for screening glutathione S-transferase-based insecticide resistance. Pesticide Biochemistry and Physiology, 68(3), 184–192. https://doi.org/10.1006/pest.2000.2512

113. Kawalek, J. C., Rew, R. S., & Heavner, J. (1984). Glutathione-S-transferase, a possible drug-metabolizing enzyme, in Haemonchus contortus: Comparative activity of a cambendazole-resistant and a susceptible strain. International Journal for Parasitology, 14(2), 173–175. https://doi.org/10.1016/0020-7519(84)90045-6

114. Zhan, B., Liu, S., Perally, S., Xue, J., Fujiwara, R., Brophy, P., Xiao, S., Liu, Y., Feng, J., Williamson, A., Wang, Y., Bueno, L. L., Mendez, S., Goud, G., Bethony, J. M., Hawdon, J. M., Loukas, A., Jones, K., & Hotez, P. J. (2005). Biochemical characterization and vaccine potential of a heme-binding glutathione transferase from the adult hookworm Ancylostoma caninum. Infection and Immunity, 73(10), 6903–6911. https://doi.org/10.1128/IAI.73.10.6903-6911.2005

115. Da Silva Vaz, I., Torino Lermen, T., Michelon, A., Sanchez Ferreira, C. A., Joaquim De Freitas, D. R., Termignoni, C., & Masuda, A. (2004). Effect of acaricides on the activity of a *Boophilus microplus* glutathione S-transferase. Veterinary Parasitology, 119(2–3), 237–245. https://doi.org/10.1016/j.vetpar.2003.11.004

116. Da Silva Vaz, I., Martinez, R. H. M., Oliveira, A., Heck, A., Logullo, C., Gonzales, J. C., Dewes, H., & Masuda, A. (1996). Functional bovine immunoglobulins in *Boophilus microplus* hemolymph. Veterinary Parasitology, 62(1–2), 155–160. https://doi.org/10.1016/0304-4017(95)00851-9

117. Raikhel, A. S., & Dhadialla, T. S. (1992). Accumulation of yolk proteins in insect oocytes. Annual Review of Entomology, 37(1), 217–251. https://doi.org/10.1146/annurev.en.37.010192.001245

118. Seixas, A., Estrela, A. B., Ceolato, J. C., Pontes, E. G., Lara, F., Gondim, K. C., & Termignoni, C. (2010). Localization and function of Rhipicephalus (Boophilus) microplus vitellin-degrading cysteine endopeptidase. Parasitology, 137(12), 1819–1831. https://doi.org/10.1017/S0031182010000624

119. Sorgine, M. H. F., Logullo, C., Zingali, R. B., Paiva-Silva, G. O., Juliano, L., & Oliveira, P. L. (2000). A heme-binding aspartic proteinase from the eggs of the hard tick *Boophilus microplus*. Journal of Biological Chemistry, 275(37), 28659–28665. https://doi.org/10.1074/jbc.M005675200

120. Logullo, C., Da Silva Vaz, I., Sorgine, M. H. F., Paiva-Silva, G. O., Faria, F. S., Zingali, R. B., De Lima, M. F. R., Abreu, L., Fialho Oliveira, E., Alves, E. W., Masuda, H., Gonzales, J. C., Masuda, A., & Oliveira, P. L. (1998). Isolation of an aspartic proteinase precursor from the egg of a hard tick, *Boophilus microplus*. Parasitology, 116(6), 525–532. https://doi.org/10.1017/S0031182098002698

121. Nascimento-Silva, M. C. L., Leal, A. T., Daffre, S., Juliano, L., da Silva Vaz, I., Paiva-Silva, G. de O., Oliveira, P. L., & Sorgine, M. H. F. (2008). BYC, an atypical aspartic endopeptidase from Rhipicephalus (Boophilus) microplus eggs. Comparative Biochemistry and Physiology - B Biochemistry and Molecular Biology, 149(4), 599–607. https://doi.org/10.1016/j.cbpb.2007.12.007

122. Bergamo Estrela, A., Seixas, A., de Oliveira Nunes Teixeira, V., Pinto, A. F. M., & Termignoni, C. (2010). Vitellin- and hemoglobin-digesting enzymes in Rhipicephalus (Boophilus) microplus larvae and females. Comparative Biochemistry and Physiology - B Biochemistry and Molecular Biology, 157(4), 326–335. https://doi.org/10.1016/j.cbpb.2010.08.002

123. Da Silva Vaz, I., Logullo, C., Sorgine, M., Velloso, F. F., Rosa De Lima, M. F., Gonzales, J. C., Masuda, H., Oliveira, P. L., & Masuda, A. (1998). Immunization of bovines with an aspartic proteinase precursor isolated from *Boophilus microplus* eggs. Veterinary Immunology and Immunopathology, 66(3–4), 331–341. https://doi.org/10.1016/S0165-2427(98)00194-9

124. Logullo, C., Moraes, J., Dansa-Petretski, M., Vaz, I. S., Masuda, A., Sorgine, M. H. F., Braz, G. R., Masuda, H., & Oliveira, P. L. (2002). Binding and storage of heme by vitellin from the cattle tick, *Boophilus microplus*. Insect Biochemistry and Molecular Biology, 32(12), 1805–1811. https://doi.org/10.1016/S0965-1748(02)00162-5

125. Seixas, A., Leal, A. T., Nascimento-Silva, M. C. L., Masuda, A., Termignoni, C., & da Silva Vaz, I. (2008). Vaccine potential of a tick vitellin-degrading enzyme (VTDCE). Veterinary Immunology and Immunopathology, 124(3–4), 332–340. https://doi.org/10.1016/j.vetimm.2008.04.001

126. Seixas, A., Oliveira, P., Termignoni, C., Logullo, C., Masuda, A., & da Silva Vaz, I. J. (2012). Rhipicephalus (Boophilus) microplus embryo proteins as target for tick vaccine. Veterinary Immunology and Immunopathology, 148(1–2), 149–156. https://doi.org/10.1016/j.vetimm.2011.05.011

127. Rivera, S., Khrestchatisky, M., Kaczmarek, L., Rosenberg, G. A., & Jaworski, D. M. (2010). Metzincin proteases and their inhibitors: Foes or friends in nervous system physiology? Journal of Neuroscience, 30(46), 15337–15357. https://doi.org/10.1523/JNEUROSCI.3467-10.2010

128. Gomiz-Rüth, F. X. (2009). Catalytic domain architecture of metzincin metalloproteases. Journal of Biological Chemistry, 284(23), 15353–15357. https://doi.org/10.1074/jbc.R800069200

129. Page-McCaw, A., Ewald, A. J., & Werb, Z. (2007). Matrix metalloproteinases and the regulation of tissue remodelling. Nature Reviews Molecular Cell Biology, 8(3), 221–233. https://doi.org/10.1038/nrm2125

130. Francischetti, I. M. B., Mather, T. N., & Ribeiro, J. M. C. (2003). Cloning of a salivary gland metalloprotease and characterization of gelatinase and fibrin(ogen)lytic activities in the saliva of the Lyme disease tick vector

Ixodes scapularis. Biochemical and Biophysical Research Communications, 305(4), 869–875. https://doi.org/10.1016/S0006-291X(03)00857-X

131. Francischetti, I. M. B., Mather, T. N., & Ribeiro, J. M. C. (2005). Tick saliva is a potent inhibitor of endothelial cell proliferation and angiogenesis. Thrombosis and Haemostasis, 94(1), 167–174. https://doi.org/10.1160/TH04-09-0566

132. Decrem, Y., Beaufays, J., Blasioli, V., Lahaye, K., Brossard, M., Vanhamme, L., & Godfroid, E. (2008a). A family of putative metalloproteases in the salivary glands of the tick Ixodes ricinus. FEBS Journal, 275(7), 1485–1499. https://doi.org/10.1111/j.1742-4658.2008.06308.x

133. Decrem, Y., Mariller, M., Lahaye, K., Blasioli, V., Beaufays, J., Zouaoui Boudjeltia, K., Vanhaeverbeek, M., Cérutti, M., Brossard, M., Vanhamme, L., & Godfroid, E. (2008b). The impact of gene knock-down and vaccination against salivary metalloproteases on blood feeding and egg laying by Ixodes ricinus. International Journal for Parasitology, 38(5), 549–560. https://doi.org/10.1016/j.ijpara.2007.09.003

134. Imamura, S., da Silva Vaz, I. J., Konnai, S., Yamada, S., Nakajima, C., Onuma, M., & Ohashi, K. (2009). Effect of vaccination with a recombinant metalloprotease from Haemaphysalis longicornis. Experimental & Applied Acarology, 48(4), 345–358. https://doi.org/10.1007/s10493-009-9245-3

135. Weiss, B. L., & Kaufman, W. R. (2004). Two feeding-induced proteins from the male gonad trigger engorgement of the female tick Amblyomma hebraeum. Proceedings of the National Academy of Sciences of the United States of America, 101(16), 5874–5879. https://doi.org/10.1073/pnas.0307529101

136. Sahli. (1985). Ornithodoros moubata: Spermateleosis and secretory activity of the sperm. Experimental Parasitology, 60(3), 383–395. https://doi.org/10.1016/0014-4894(85)90045-1

137. Kamau, L., Skilton, R. A., Odongo, D. O., Mwaura, S., Githaka, N., Kanduma, E., Obura, M., Kabiru, E., Orago, A., Musoke, A., & Bishop, R. P. (2011). Differential transcription of two highly divergent gut-expressed Bm86 antigen gene homologues in the tick *Rhipicephalus appendiculatus* (Acari: Ixodida). Insect Molecular Biology, 20(1), 105–114. https://doi.org/10.1111/j.1365-2583.2010.01043.x

138. Parizi, Luís F, Reck, J., Oldiges, D. P., Guizzo, M. G., Seixas, A., Logullo, C., Oliveira, P. L. De, Termignoni, C., Martins, J. R., & Vaz, S. (2012b). Multi-antigenic vaccine against the cattle tick Rhipicephalus (Boophilus) microplus : A field evaluation. Vaccine, 30(48), 6912–6917. https://doi.org/10.1016/j.vaccine.2012.08.078

139. de la Fuente, J., Contreras, M., Estrada-peña, A., & Cabezas-cruz, A. (2017). Targeting a global health problem: Vaccine design and challenges for the control of tick-borne diseases. 1–6. https://doi.org/10.1016/j.vaccine.2017.07.097

140. Muhanguzi, D. (2022). Anti-Tick Vaccines: Current Advances and Future Prospects. In Thomas S. (Ed.), Vaccine Design. Methods in Molecular Biology, vol 2411. (Second, pp. 253–267). Humana, New York, NY. https://doi.org/https://doi.org/10.1007/978-1-0716-1888-2_15

141. Sette, A., & Rappuoli, R. (2010). Reverse vaccinology: Developing vaccines in the era of genomics. Immunity, 33(4), 530–541. https://doi.org/10.1016/j.immuni.2010.09.017

142. de la Fuente, José, & Contreras, M. (2021). Vaccinomics: a future avenue for vaccine development against emerging pathogens. Expert Review of Vaccines, 0(0). https://doi.org/10.1080/14760584.2021.1987222

143. Haralambieva, I. H., & Poland, G. A. (2010). Vaccinomics, predictive vaccinology and the future of vaccine development. Future Microbiology, 5(12), 1757–1760. https://doi.org/10.2217/fmb.10.146

144. Poland, G. A., Ovsyannikova, I. G., Kennedy, R. B., Haralambieva, I. H., & Jacobson, R. M. (2011). Vaccinomics and a new paradigm for the development of preventive vaccines against viral infections. OMICS A Journal of Integrative Biology, 15(9), 625–636. https://doi.org/10.1089/omi.2011.0032

145. Poland, G. A., Kennedy, R. B., McKinney, B. A., Ovsyannikova, I. G., Lambert, N. D., Jacobson, R. M., & Oberg, A. L. (2013). Vaccinomics, adversomics, and the immune response network theory: Individualized vaccinology in the 21st century. Seminars in Immunology, 25(2), 89–103. https://doi.org/10.1016/j.smim.2013.04.007

146. Flower, D. R. (2009). Bioinformatics for Vaccinology. In Bioinformatics for Vaccinology. https://doi.org/10.1002/9780470699836

147. Artigas-Jerónimo, S., Pastor Comín, J. J., Villar, M., Contreras, M., Alberdi, P., Viera, I. L., Soto, L., Cordero, R., Valdés, J. J., Cabezas-Cruz, A., Estrada-Peña, A., & de la Fuente, J. (2020). A novel combined scientific and artistic approach for the advanced characterization of interactomes: The akirin/subolesin model. Vaccines, 8(1), 1–27. https://doi.org/10.3390/vaccines8010077

148. de la Fuente, José, Maritz-Olivier, C., Naranjo, V., Ayoubi, P., Nijhof, A. M., Almazán, C., Canales, M., de la Lastra, J. M. P., Galindo, R. C., Blouin, E. F., Gortazar, C., Jongejan, F., & Kocan, K. M. (2008). Evidence of the role of tick subolesin in gene expression. BMC Genomics, 9, 1–16. https://doi.org/10.1186/1471-2164-9-372

149. Kasaija, P. D., Contreras, M., Kabi, F., Mugerwa, S., & de la Fuente, J. (2020). Vaccination with recombinant subolesin antigens provides cross-tick species protection in bos indicus and crossbred cattle in Uganda. Vaccines, 8(2), 1–17. https://doi.org/10.3390/vaccines8020319

150. Mbulaiteye-Saimo, M., Odongo, Mwaura, Bishop, Vlak, Musoke, Lubega, Van Oers, & George W. Lubega. (2011). Recombinant *Rhipicephalus appendiculatus* gut (Ra86) and salivary gland cement (Trp64) proteins as candidate antigens for inclusion in tick vaccines: protective effects of Ra86 on infestation with adult R. appendiculatus. Vaccine: Development and Therapy, 15. https://doi.org/10.2147/vdt.s20827

151. Kamau, L. M. (2005). Isolation, recombinant expression and characterization of polymorphism of BM86 vaccine antigen homologues from *rhipicephalus appendiculatus* (Doctoral dissertation, Kenyatta University).

152. Dogo, G., Kwaga, J., Umoh, J., Agbede, R., & Jongejan, F. (2015). Molecular Detection and Characterization of Bm86 Gene Homologues from Hyalomma truncatum, Rhipicephalus (Boophilus) annulatus and Rhipicephalus (Boophilus) decoloratus for the Development of an Anti-Tick Vaccine in Nigeria. *International Journal of Livestock Research*, *5*(4), 34. https://doi.org/10.5455/ijlr.20150410031140

153. Ben Said, M. (2015). Molecular and Phylogenetic Study of Bm86 Gene Ortholog from Hyalomma excavatum Tick from Tunisia: Taxonomic and Immunologic Interest. *Hereditary Genetics*, *04*(03). https://doi.org/10.4172/2161-1041.1000154

154. Republic of Uganda. The presidential technical advisory committee on the tick resistance challenge. In Strategy for Efficient, Effective and Sustainable Management of Ticks, Acaricide Resistance and Tick-Borne Diseases in Uganda; Republic of Uganda: Kampala, Uganda, 2017.

155. Lodos, Jorge, Boue, O., & De, J. (2000). A model to simulate the effect of vaccination against Boophilus ticks on cattle. 87, 315–326.

156. Lodos, J, Ochagavia, M. E., Rodriguez, M., & Fuente, J. D. La. (1999). A simulation study of the effects of acaricides and vaccination on Boophilus cattle tick populations. 38, 47–63.

157. Perry, B. D., & Young, A. S. (1995). The past and future roles of epidemiology and economics in the control of tick-borne diseases of livestock in Africa: the case of theileriosis. 25(95), 107–120.

158. Irvin, A. D., Mcdermott, J. J., & Perry, B. D. (1996). Epidemiology of Ticks and Tick-borne Diseases in Eastern, Central and Southern Africa. In Africa (Issue March).
